# A Case of Solitary Fibrous Tumor with Tumor Rupture during the Waiting Period for Elective Surgery

**DOI:** 10.70352/scrj.cr.26-0429

**Published:** 2026-08-28

**Authors:** Atomu Kiriyama, Saseem Poudel, Hironobu Takano, Hideyuki Wada, Takeo Nitta, Soichi Murakami, Toshiaki Shichinohe, Toshiaki Takahashi, Kanako C Hatanaka, Satoshi Hirano

**Affiliations:** 1 Department of Gastroenterological Surgery II, Faculty and Graduate School of Medicine, Hokkaido University, Sapporo, Hokkaido, Japan; 2 Department of Surgical Pathology, Hokkaido University Hospital, Sapporo, Hokkaido, Japan; 3 Center for Development of Advanced Diagnostics, Hokkaido University Hospital, Sapporo, Hokkaido, Japan

**Keywords:** solitary fibrous tumor, tumor rupture, peritonitis, intra-abdominal tumor, signal transducer and activator of transcription 6

## Abstract

**INTRODUCTION:**

Solitary fibrous tumor (SFT) is a rare mesenchymal neoplasm that most commonly arises in the pleura but can occur at virtually any anatomical site, including the abdominal cavity. Although rupture of SFT has occasionally been reported, most such cases involve intrathoracic tumors presenting with hemorrhage, and peritonitis caused by rupture of an intra-abdominal SFT is exceedingly rare.

**CASE PRESENTATION:**

A man in his 70s was referred to Hokkaido University Department of Gastroenterological Surgery II after contrast-enhanced CT revealed a 12 × 9-cm heterogeneous pelvic mass with poor enhancement accompanied by ascites. Endoscopic US–guided fine-needle aspiration had been performed at the previous hospital but failed to yield a definitive diagnosis, and elective tumor resection was planned. One week after the initial visit, the patient was brought to our hospital with abdominal pain. Emergency CT showed slight enlargement of the tumor, intratumoral fluid collection, and increased fat stranding around the tumor. Conservative management with intravenous antibiotics was initiated because there were no overt signs of generalized peritonitis. However, inflammatory markers worsened and fever developed, prompting emergency surgery on hospital day 2. Laparotomy revealed partial rupture of the tumor wall with leakage of contaminated intratumoral fluid into the peritoneal cavity. The tumor pedicle was identified at the base of the sigmoid mesocolon, and complete resection was achieved. The operative time was 124 min, and the estimated blood loss was 1100 mL. Histopathological examination revealed a patternless proliferation of spindle cells within a fibrous stroma with staghorn-like vasculature. Immunohistochemistry showed nuclear positivity for signal transducer and activator of transcription 6 and positivity for CD34, confirming the diagnosis of SFT. According to the modified Demicco model, the tumor was classified as intermediate risk. The patient was discharged on POD 31.

**CONCLUSIONS:**

When managing a large intra-abdominal tumor whose growth rate cannot be reliably assessed, the possibility of rupture should be kept in mind, and emergency surgery should not be delayed when abdominal findings worsen. Although the prognostic impact of tumor rupture in SFT remains unclear, this event may justify closer postoperative surveillance than that indicated by existing risk classification schemes.

## Abbreviations

CA19-9
: carbohydrate antigen 19-9
CEA
: carcinoembryonic antigen
CRP
: C-reactive protein
EUS-FNA
: endoscopic US–guided fine-needle aspiration
GIST
: gastrointestinal stromal tumor
PSA
: prostate-specific antigen
SFT
: solitary fibrous tumor
STAT6
: signal transducer and activator of transcription 6
WBC
: white blood cell

## INTRODUCTION

SFT is a rare mesenchymal neoplasm that occurs predominantly in the pleura but may arise at various extrapleural sites, including the abdominal cavity.^1–4)^ Preoperative diagnosis is often challenging, and histopathological examination is usually required for a definitive diagnosis.^4–6)^ Although rare, rupture of SFT has also been reported, mostly involving intrathoracic tumors.^7–13)^ In contrast, peritonitis caused by rupture of an intra-abdominal SFT is extremely rare. Herein, we report a case of intra-abdominal SFT that ruptured during the waiting period for elective surgery and resulted in peritonitis, together with a review of the relevant literature.

## CASE PRESENTATION

A man in his 70s with a medical history of hypertension presented to a previous hospital with constipation. During the diagnostic workup, CT revealed a pelvic mass. EUS-FNA was performed. According to the referral documentation from the previous hospital, EUS-FNA was performed via a transrectal approach using a 22-gauge needle, with 3 needle passes. The referral report described only a mass visible within the pelvic cavity, and it remains unclear whether the tumor was presumed at that time to originate from the rectal wall itself. However, the previous hospital’s pathology report indicated extensive necrosis that precluded evaluation of the overall tissue architecture, and a definitive diagnosis could not be established. Immunohistochemistry performed on that specimen was positive for CD34 and negative for PSA, p504s, CEA, S-100 protein, α-smooth muscle actin, myoglobin, HMB-45, c-kit, and DOG1. Compared with CT findings obtained at the previous hospital, MRI and PET-CT performed at our hospital demonstrated interval enlargement of the tumor. He was therefore referred to Hokkaido University Department of Gastroenterological Surgery II for further evaluation and management, and surgical resection was planned for both diagnostic and therapeutic purposes.

**Fig. 1:**
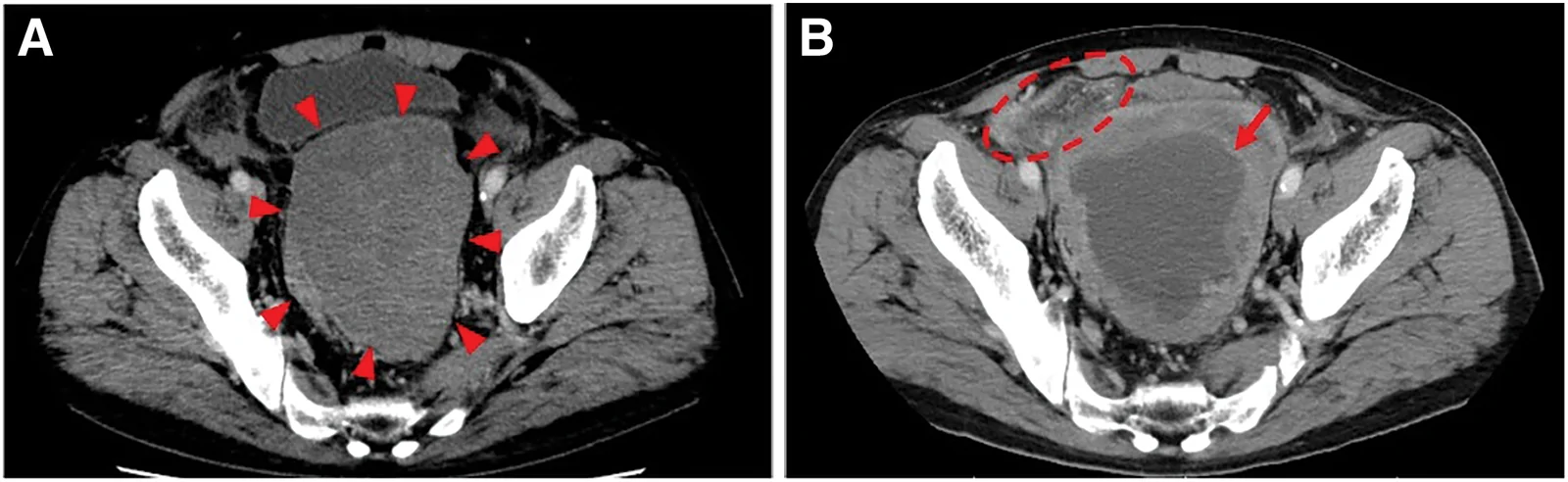
CT findings. (**A**) Preoperative contrast-enhanced CT obtained at the previous hospital 5 weeks ago showed a well-defined heterogeneous pelvic mass measuring 12 × 9 cm with poor enhancement (arrowheads) and associated ascites. (**B**) Emergency CT obtained 1 week later showed slight enlargement of the tumor, newly developed intratumoral fluid collection (arrow), and increased fat stranding around the tumor (dotted circle).

At the initial visit to Hokkaido University Department of Gastroenterological Surgery II, laboratory tests showed a WBC count of 10400/μL and a CRP level of 9.32 mg/dL. Tumor markers, including CEA and CA19-9, were within normal limits. Contrast-enhanced CT taken at the previous hospital 5 weeks ago demonstrated a well-defined heterogeneous pelvic mass measuring 12 × 9 cm with poor enhancement, accompanied by ascites (**Fig. 1A**).

One week after the initial visit, the patient was brought emergently to our hospital because of abdominal pain. On arrival, his vital signs were as follows: body temperature, 36.0°C; blood pressure, 104/59 mmHg; heart rate, 80 beats/min; and peripheral oxygen saturation, 95% on room air. Physical examination revealed tenderness in the lower abdomen without guarding or rebound tenderness. Laboratory tests showed a WBC count of 9000/μL and a CRP level of 12.81 mg/dL, which were similar to those at the initial visit. Emergency CT revealed that the pelvic tumor had slightly enlarged to 13 × 9.5 cm and now contained intratumoral fluid collection, with increased fat stranding around the tumor. Ascites was present but had not markedly increased compared with the initial CT. No free air was observed (**Fig. 1B**).

Although CT findings suggested possible intratumoral fluid leakage, carcinomatous peritonitis could not be excluded as an alternative explanation for the ascites and peritoneal changes. Given the absence of overt generalized peritonitis and the diagnostic uncertainty, conservative management with intravenous piperacillin/tazobactam was initiated. However, inflammatory markers continued to worsen, and fever developed during the night of hospital day 1. Conservative treatment was considered ineffective, and emergency surgery was performed on hospital day 2.

**Fig. 2:**
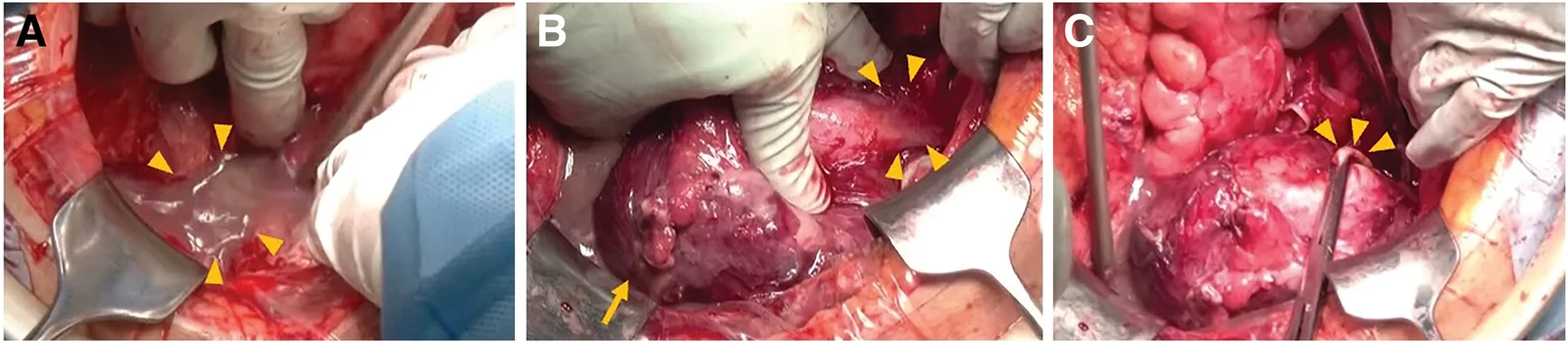
Intraoperative findings. (**A**) Partial rupture of the tumor wall with leakage of contaminated intratumoral fluid into the peritoneal cavity (arrowheads). (**B**) The tumor was identified (arrow) and its pedicle was traced to the base of the sigmoid mesocolon (arrowheads). (**C**) The tumor was resected after ligation and division of the pedicle (arrowheads).

Under general anesthesia, bilateral ureteral stents were placed before laparotomy. On entering the abdominal cavity, partial rupture of the tumor wall was identified, with leakage of contaminated fluid from inside the tumor into the peritoneal cavity (**Fig. 2A**). The tumor pedicle originated from the base of the sigmoid mesocolon, with adhesion to the bowel wall but no evidence of transmural invasion; complete separation was achieved by blunt dissection without bowel resection and the pedicle was ligated and divided (**Fig. 2B** and **2C**). The operative time was 124 min, and the estimated blood loss was 1100 mL, including intratumoral contents.

Postoperatively, inflammatory markers remained elevated for several days but gradually improved with antibiotic therapy. Intraoperative peritoneal fluid cultures yielded *Eggerthella lenta* and *Bacteroides uniformis*, confirming polymicrobial bacterial contamination of the peritoneal cavity. Blood cultures drawn on hospital day 1 at the time of fever onset yielded *Eggerthella lenta* from 1 anaerobic bottle, suggesting possible bacteremia, although contamination could not be excluded given the single positive set. The patient was discharged home on POD 31. He was not given chemotherapy and was scheduled for outpatient close follow-up every 3 months, with chemotherapy to be considered only if a recurrence occurred. He is currently 9 months post-surgery and shows no signs of recurrence.

**Fig. 3:**
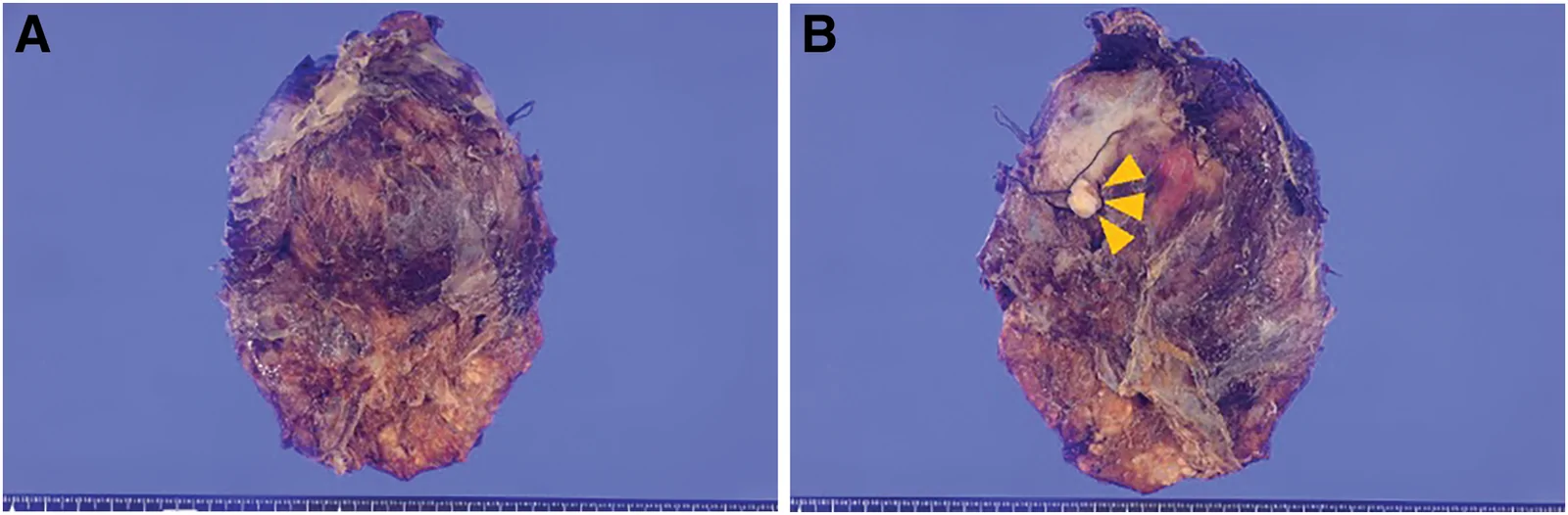
Gross findings of the resected specimen. (**A**) The resected specimen was a cystic mass measuring 15.0 × 9.3 × 4.3 cm, with a heterogeneous cut surface composed of white nodules, myxoid degeneration, hemorrhage, and necrosis. (**B**) The tumor pedicle originating from the sigmoid mesocolon is indicated (arrowheads).

Gross examination of the resected specimen revealed a cystic mass measuring 15.0 × 9.3 × 4.3 cm with a heterogeneous cut surface composed of white nodules, myxoid degeneration, hemorrhage, and necrosis in varying proportions (**Fig. 3A** and **3B**).

**Fig. 4:**
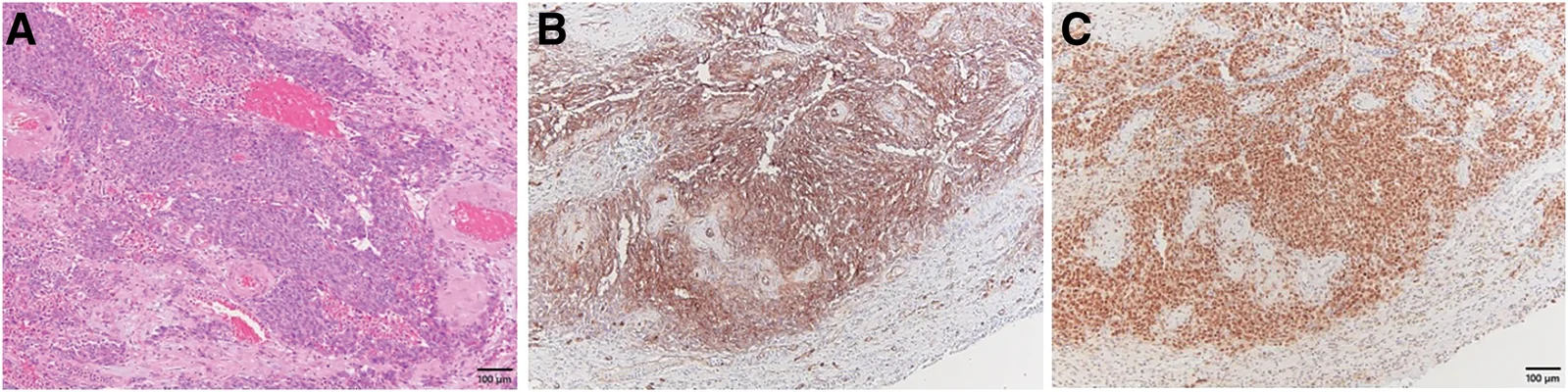
Histopathological and immunohistochemical findings. (**A**) Hematoxylin and eosin staining showed spindle cell proliferation with a patternless architecture in a fibrous stroma and staghorn-like vasculature. (**B**) Immunohistochemistry showed positivity for CD34. (**C**) Immunohistochemistry showed nuclear positivity for STAT6. STAT6, signal transducer and activator of transcription 6

Histopathological examination revealed spindle to short spindle-shaped tumor cells with mildly hyperchromatic oval nuclei and a high nuclear-to-cytoplasmic ratio. These cells showed a patternless architecture within an abundant fibrous stroma containing dilated staghorn-like vessels (**Fig. 4A**). No mitotic figures were identified (0/10 high-power fields), and infiltrative growth was absent. Extensive necrosis and myxoid degeneration were present, although the precise proportion of necrosis could not be determined on pathological examination.

Immunohistochemically, the tumor cells were positive for CD34 and nuclear STAT6 (**Fig. 4B** and **4C**) and negative for S-100 protein, SOX10, desmin, α-smooth muscle actin, CD31, ERG, MDM2, and CDK4. c-kit and DOG1 immunohistochemistry, which had already been performed on the EUS-FNA specimen at the previous hospital and was negative, was not repeated on the resected specimen at our institution. On the basis of these findings, the tumor was diagnosed as SFT. The Ki-67 labeling index was 10%–15% in hot spots. According to the modified Demicco model, the tumor was classified as intermediate risk.

## DISCUSSION

We report an extremely rare case of SFT that ruptured during the waiting period for elective surgery. This case highlights 2 clinical issues in the management of large intra-abdominal mesenchymal tumors: the difficulty of establishing a preoperative tissue diagnosis, and the potential for unpredictable rupture even in tumors generally regarded as slow-growing, whose contributing factors—such as spontaneous wall breakdown, rapid enlargement, and procedural injury—are often difficult to disentangle.

SFT is diagnosed histopathologically, showing a patternless proliferation of spindle cells with staghorn-like vasculature, together with diffuse CD34 positivity and nuclear STAT6 expression; the latter reflects the underlying NAB2–STAT6 gene fusion and serves as a highly sensitive and specific marker.^14–16)^ However, CD34 positivity alone is not specific to SFT, and a broader immunohistochemical panel is therefore required to exclude GIST, peripheral nerve sheath tumors, smooth muscle and skeletal muscle tumors, PEComa, and metastatic carcinoma.^17)^ In the present case, the EUS-FNA specimen obtained at the previous hospital was positive for CD34 and negative for all of the corresponding markers, effectively excluding these differential diagnoses. However, STAT6 immunostaining was not performed and the extensive necrosis in the specimen precluded assessment of the characteristic patternless architecture, so a definitive diagnosis of SFT could not be established preoperatively. Because there are no pathognomonic radiological features on CT imaging,^6)^ differentiation from other mesenchymal tumors, such as GIST, is often difficult before surgery, and nuclear STAT6 positivity with the patternless architecture was confirmed only on the resected specimen. Importantly, this diagnostic failure reflected an intrinsic feature of the tumor rather than an insufficient specimen volume or a technical failure of the procedure: the same extensive necrosis was present in the resected specimen, making it unlikely that a repeat EUS-FNA would have yielded architecturally preserved tissue. This case therefore underscores an inherent limitation of needle biopsy for large mesenchymal tumors with extensive internal necrosis, and suggests that, when clinical deterioration is observed, delaying surgery until a definitive diagnosis is established may not be appropriate.

Regarding the procedure itself, pelvic mesenchymal tumors—including those arising from the sigmoid mesocolon—often lie in close proximity to the rectum and are difficult to distinguish from rectal-wall lesions on cross-sectional imaging, so a transrectal approach is commonly selected for pelvic masses accessible via the rectum regardless of their organ of origin; whether the tumor was specifically presumed to arise from the rectum in this case cannot be determined from the available referral information. Needle-tract tumor seeding is a genuine, if uncommon, risk of any transluminal biopsy of a mesenchymal tumor and cannot be excluded in the present case. Nevertheless, given the substantial difficulty of diagnosing large pelvic mesenchymal tumors on imaging alone and the differing therapeutic implications of SFT versus other entities such as GIST, EUS-FNA remains a reasonable first-line diagnostic strategy; the cited literature reports elevated rates of bleeding and infection with the transrectal approach but does not identify it as contraindicated for pelvic masses.^18,19)^ Clinicians should therefore weigh the diagnostic benefit of EUS-FNA against its inherent risks—including tumor seeding, hemorrhage, and, as suggested by the present case, a possible contribution to tumor rupture—particularly when the mass is large and its organ of origin cannot be determined preoperatively.

The cause of rupture cannot be definitively determined, and multiple mechanisms warrant consideration. First, spontaneous rupture secondary to intratumoral necrosis, hemorrhage, or cystic degeneration is plausible, given that the resected specimen demonstrated extensive necrosis and myxoid degeneration—findings commonly observed in large SFTs.^6)^ Second, rapid tumor enlargement may have increased intratumoral pressure and contributed to wall disruption. Third, EUS-FNA performed at the previous hospital approximately 6 weeks before rupture may have played a role; transrectal EUS-FNA carries serious adverse event rates as high as 5.6%.^18,19)^ Physical injury from needle puncture combined with potential bacterial inoculation via the transrectal route may have predisposed the tumor to secondary infection and subsequent rupture. The site of rupture identified intraoperatively was located on the peritoneal surface of the tumor, anatomically consistent with a transrectal puncture approach. Although a needle-tract defect could not be confirmed on intraoperative or pathological examination, this does not exclude EUS-FNA as a contributing factor. Causality cannot be established, and the rupture most likely resulted from a combination of these factors. Notably, rather than tumor enlargement per se, the interval development of intratumoral fluid collection and increased peritumoral fat stranding on CT may represent more clinically meaningful signs of impending rupture. These findings likely reflect progressive intratumoral necrosis, hemorrhage, and cystic degeneration; clinicians should therefore remain alert to such interval changes even in the absence of marked size progression.

Rupture of SFT itself is a rare event. A literature search was performed using PubMed and Ichushi-Web (Japan Medical Abstracts Society) with the search terms “solitary fibrous tumor” AND “rupture” in PubMed, and the corresponding Japanese terms in Ichushi-Web, without date restrictions, up to July 4, 2026. Because case reports of rare tumors are often published in local-language journals not indexed in PubMed, Ichushi-Web was additionally searched to ensure comprehensive case ascertainment, while the PubMed search ensured inclusion of the international English-language literature. Additional cases were identified through manual review of reference lists. All case reports describing rupture of SFT, regardless of tumor location, were included, yielding a total of 11 cases (**Table 1**).^7–13,20–23)^ Most involved intrathoracic tumors presenting with hemorrhage. As shown in **Table 1**, only 2 cases of ruptured intra-abdominal SFT have been reported in Japan, and reports from other countries are also scarce. As in the present case, the possibility of rupture must always be considered when managing large intra-abdominal tumors whose growth rate cannot be reliably assessed.

**Table 1:** Summary of case reports of ruptured SFTs

Author	Year	Age	Sex	Origin	Tumor size (max)	Rupture mode	Pathology
Asai^7)^	2003	30s	Male	Pleura	9 cm	Hemothorax	Benign
Kristensen^8)^	2007	30s	Male	Pleura	11 cm	Hemothorax	Benign
Sasaki^20)^	2009	20s	Male	Perisplenic tissue	5 cm	Intraperitoneal bleeding	Malignant
Shigemitsu^9)^	2011	50s	Male	Pleura	10 cm	Hemothorax	Malignant
Kyogoku^21)^	2013	30s	Male	Ileocecal mesocolon	22 cm	Peritonitis	Benign
Morita^22)^	2013	30s	Male	Posterior mediastinum	7 cm	Hemothorax	Benign
Shao^10)^	2014	40s	Female	Pleura	3 cm	Hemothorax	Benign
Negri^11)^	2014	30s	Female	Pleura	7 cm	Hemothorax	Benign
Tan^12)^	2016	70s	Female	Pleura	9.7 cm	Hemothorax	Benign
Komatsu^13)^	2024	40s	Male	Pleura	5 cm	Hemothorax	Benign
Naravejsakul^23)^	2025	50s	Male	Kidney	23.8 cm	Spontaneous	Malignant
Our case	2026	70s	Male	Sigmoid mesocolon	15 cm	Peritonitis	Intermediate

SFT, solitary fibrous tumor

Tumor rupture may also have prognostic implications. Risk in SFT is commonly stratified using the modified Demicco model,^24)^ which incorporates age, tumor size, mitotic activity, and necrosis. In the present case, the tumor was classified as intermediate risk, for which the reported 10-year metastasis rate is approximately 7%. However, the prognostic impact of tumor rupture in SFT remains unknown, and no evidence confirms that it carries the adverse significance recognized in GIST, where rupture is an independent adverse prognostic factor^25,26)^; the parallel is therefore conceptual rather than evidence-based. Nevertheless, intra-abdominal contamination with tumor contents raises a theoretical concern for peritoneal dissemination that existing risk models do not capture. Although postoperative surveillance guidelines specific to SFT have not yet been established,^27)^ late recurrence more than 10 years after resection has been reported,^3,28)^ emphasizing the need for long-term follow-up. As a practical approach following general soft tissue sarcoma surveillance protocols, imaging every 3–4 months for the first 2–3 years, every 6 months until 5 years, and annually thereafter has been recommended.^29)^ A review of pleural SFT recommended CT every 6 months for the first 2 years and annually thereafter.^30)^ Given the intermediate-risk classification compounded by rupture with intra-abdominal contamination, closer surveillance than that used for standard intermediate-risk SFT may be a reasonable precaution, although with only 9 months of follow-up no conclusion regarding this patient’s recurrence risk can yet be drawn. As adjuvant chemotherapy following resection has not been established for SFT,^28,29)^ close surveillance with systemic therapy reserved for recurrence was considered the most appropriate strategy in this case.

## CONCLUSIONS

We report a case of SFT that ruptured during the waiting period before elective surgery.

Preoperative differentiation of intra-abdominal tumors can be challenging. When the growth rate of a large tumor is difficult to assess, it is essential to remain alert to the possibility of tumor rupture and not to hesitate in proceeding with emergency surgery based on changes in abdominal findings.

## DECLARATIONS

### Availability of data and materials

Data sharing is not applicable to this article because no datasets were generated or analyzed during the current study.
